# Facile Exfoliation for High-Quality Molybdenum Disulfide Nanoflakes and Relevant Field-Effect Transistors Developed With Thermal Treatment

**DOI:** 10.3389/fchem.2021.650901

**Published:** 2021-04-26

**Authors:** Yu Zhang, Xiong Chen, Hao Zhang, Shaozu Hu, Guohong Zhao, Meifang Zhang, Wei Qin, Zhaohua Wang, Xiaowei Huang, Jun Wang

**Affiliations:** ^1^Organic Optoelectronics Engineering Research Center of Fujian's Universities, College of Electronics and Information Science, Fujian Jiangxia University, Fuzhou, China; ^2^Fujian Innovation Center of Additive Manufacturing, Fuzhou, China; ^3^College of Science, Shanghai Institute of Technology, Shanghai, China

**Keywords:** MoS_2_, exfoliation, thermal treatment, surface free energy, van de Waals force, FET

## Abstract

Molybdenum disulfide (MoS_2_), a typical member of the transition metal dichalcogenides (TMDs) group, is known for its excellent electronic performance and is considered a candidate next-generation semiconductor. The preparation of MoS_2_ nanoflakes for use as the core of semiconducting devices depends on mechanical exfoliation, but its quality has not yet been optimized. In this paper, a novel exfoliation method of achieving MoS_2_ nanoflakes is proposed. We find that the size and yield of the exfoliated flakes are improved after thermal treatment for 2 h at a temperature of 110°C followed by precooling for 10 min in ambient air. The new method has the advantage of a 152-fold larger size of obtained MoS_2_ flakes than traditional mechanical exfoliation. This phenomenon may be attributable to the differences in van Der Waals force and the increase in surface free energy at the interface induced by thermal treatment. In addition, a field-effect transistor (FET) was fabricated on the basis of multilayer MoS_2_ prepared according to a new process, and the device exhibited a typical depleted-FET performance, with an on/off ratio of ~10^5^ and a field-effect mobility of 24.26 cm^2^/Vs in the saturated region when *V*_G_ is 10 V, which is generally consistent with the values for devices reported previously. This implies that the new process may have potential for the standard preparation of MoS_2_ and even other 2D materials as well.

## Introduction

The era of two-dimensional (2D) materials was initiated with the birth of graphene exfoliated from graphite using Scotch tape by Novoselov et al. (2004). These materials incorporate flake-like structures with a lateral size from 100 nm up to a few micrometers and a thickness that is typically on the scale of a nanometer, a single or only a few layers of atoms (Zhang, 2015). Over the last decade, more members of the 2D-material family, including black phosphorus (Cartz et al., 1979; Zhang X. et al., 2015), hexagonal boron nitride (Lin et al., 2010; Weng et al., 2016), graphitic carbon nitride (g-C_3_N_4_) (Zhang J. et al., 2015; Ong et al., 2016), and transition metal dichalcogenides (TMDs) (Huang et al., 2013; Lv et al., 2015; Tan and Zhang, 2015) have been developed and explored in depth.

A TMDs is a type of 2D material that consists of a transition metal element (e.g., Mo, W) and a chalcogen (e.g., S, Se) (Wilson and Yoffe, 1969; Beal et al., 1972; Qiu et al., 2012). As a semiconductor material for TMDs, molybdenum disulfide (MoS_2_) is known for its special direct–indirect bandgap widths of 1.8 and 1.3 eV, respectively (Mak et al., 2012), high field-effect mobility (Radisavljevic et al., 2011) in electronic devices, and inner structure of single or multiple atomic layers, which may exhibit significant promise in the application of high-performance, flexible electronic devices.

The first step in preparing devices based on MoS_2_ is to transfer the material to certain substrates. The methods for this can be divided into two categories, namely, “top to bottom” and “bottom to top.” The process for the former is mainly executed by making the bulk into flake through methods such as micromechanical exfoliation (Novoselov et al., 2004; Nicolosi et al., 2013; Li et al., 2014; Yi and Shen, 2015; Niu et al., 2016) and liquid exfoliation assisted by ion intercalation (Dines, 1975; Joensen et al., 1986). The latter is achieved by depositing the atomic layer on the substrate using one of several techniques, including chemical vapor deposition (Liu et al., 2005; Wu et al., 2013) and atomic-layer deposition (Riikka, 2005; Jin et al., 2014). Mechanical exfoliation is currently considered a relatively ideal way to achieve nanoflakes of 2D materials for a free-contamination, crystalline product in a way that is independent of equipment. However, due to the non-standardized and uncontrollable process used, high-quality 2D nanoflakes with large areas and yields are more difficult to obtain using mechanical exfoliation. Some studies have been conducted to improve this process. For example, Desai et al. demonstrated an exfoliation technique using evaporated gold films to exfoliate large-area TMD monolayers onto various substrates, such as SiO_2_/Si and quartz (Desai et al., 2016). Huang et al. introduced a universal Au-assisted method of mechanical exfoliation and demonstrated its effectiveness by exfoliating MoS_2_ monolayers (Huang et al., 2020). However, these methods must be carried out with some other materials, which have to be removed with solvents, leading to contamination at the interface. Furthermore, multilayer TMDs offer higher drive currents from multiple conducting channels than monolayer TMD channels, with a higher density of states as well as lower sensitivity to charge impurities at the interface, better immunity against noise in air, and greater ability to form large-size nanoflakes (Kim et al., 2012; Kwon H. et al., 2014; Kwon H. J. et al., 2014).

In this work, we report a method for preparing MoS_2_ nanoflakes based on mechanical exfoliation through thermal treatment without assistance. In this way, the values for the number of samples that reach a specific size were executed to estimate the effects and achieve ideal parameters for the new process. Subsequently, we characterized the flake samples according to Raman spectra and atomic force microscopy (AFM). We confirmed that thermal treatment can clearly improve the size and yield of MoS_2_ nanoflakes. This approach is a prominent means of preparing 2D-layered materials. Furthermore, to explore the application of this novel means of materials preparation, a field-effect transistor (FET) was fabricated, using multilayer-thick MoS_2_ flakes exfoliated after thermal treatment. The field-effect mobility of the device is 24.26 cm^2^/Vs in the saturated region, and the on/off ratio is ~10^5^. These results are consistent with those of devices reported previously, which implies the applicability of the new nanoflake-preparation method.

## Materials and Methods

MoS_2_ was purchased in bulk from a 2D semiconductor manufacturer. The substrates were formed from two parts. The upper was 300 nm thermal oxygenized SiO_2_, and the lower was highly doped n-type Si. The Scotch tape used to dissociate the bulk into flakes was purchased from a 3M Company.

**Figure 1:**
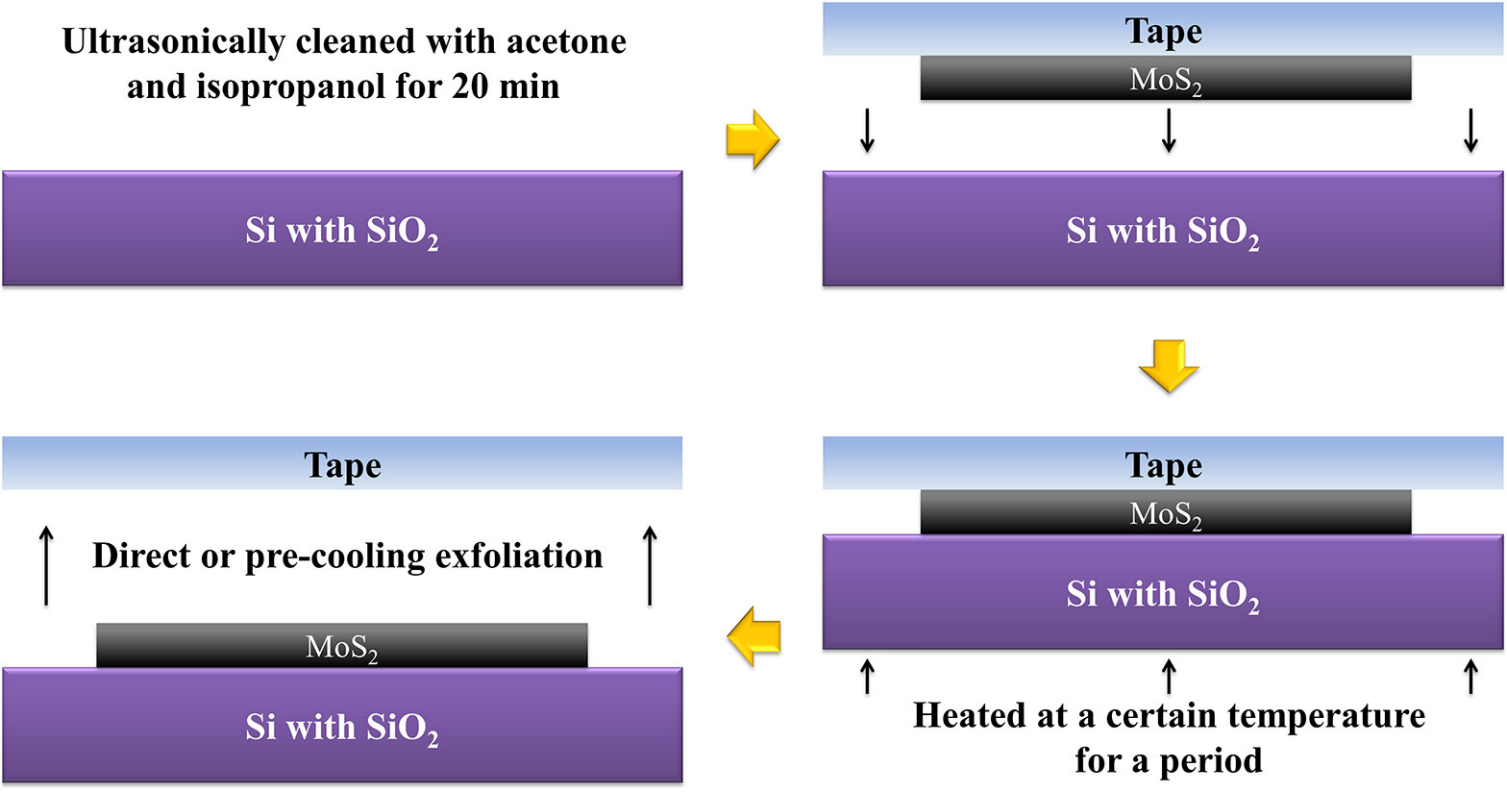
Preparation of MoS_2_ nanoflakes by thermal treatment.

The MoS_2_ nanoflakes were prepared as follows (Figure 1). First, the substrate was ultrasonically cleaned with acetone and isopropanol for 20 min to reduce contamination. Second, we reduced the bulk of the MoS_2_ by transferring it from the original tape to four others. The last transferred tape was placed to cover the substrate, and the MoS_2_ was in contact with the SiO_2_ layer. We repeatedly pressed the surface of the tape with the thumb for about 20 s to ensure relatively close contact. Then thermal treatment was carried out under the wafer for a period of time. Eventually, we removed the tapes from the substrate using direct or precooling exfoliation.

Following the above procedures, we utilized MoS_2_ nanoflakes on Si/SiO_2_ substrates as an active layer and Ag as drain-source electrodes to fabricate FETs. Here, the drain-source electrodes were formed using shadow masking and vacuum vapor deposition.

**Figure 2:**
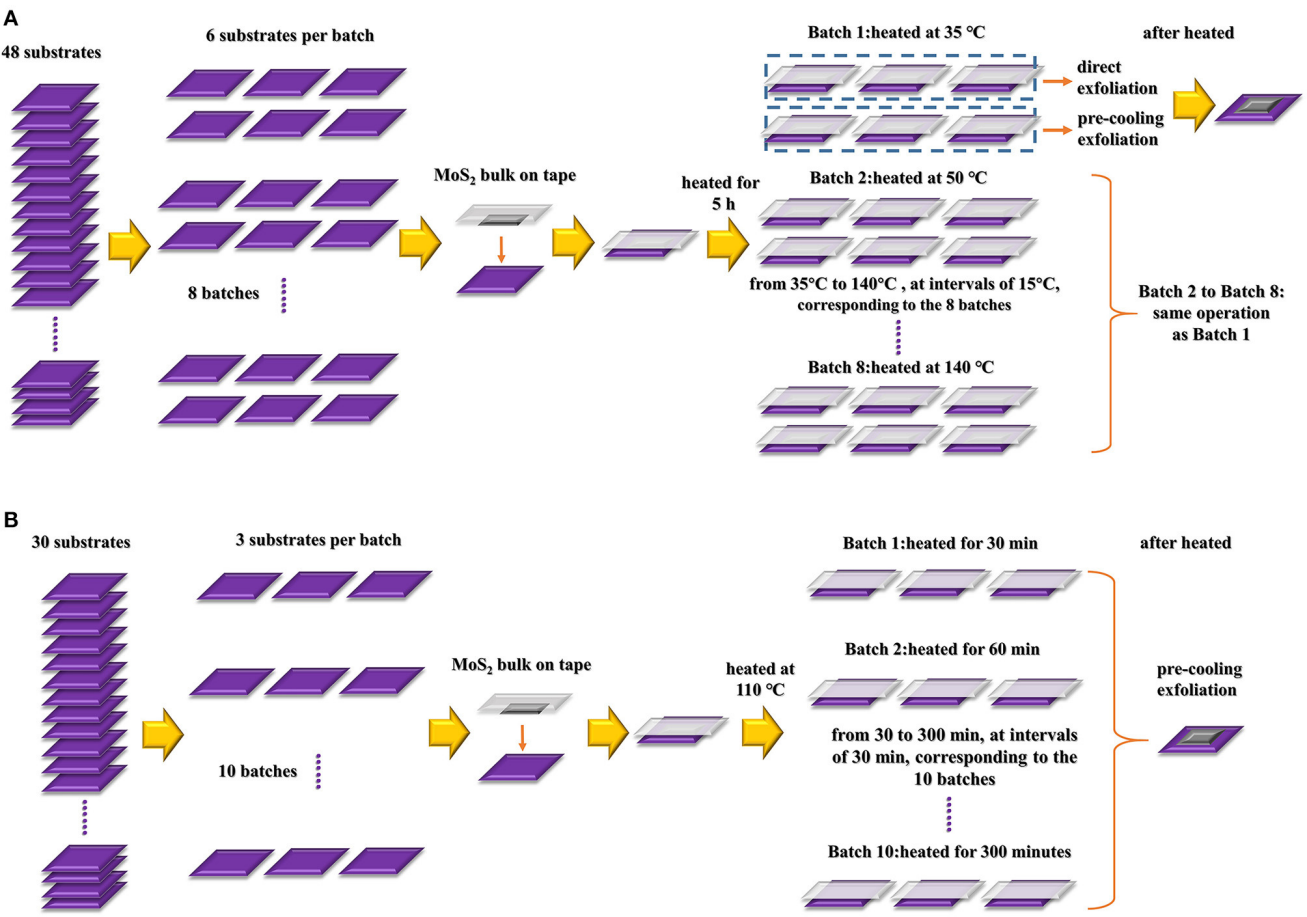
Processes of **(A)** temperature-dependent and **(B)** time-dependent experiments.

To explore the influence of temperature on the size and quantity of the flake product, we prepared 48 Si/SiO_2_ substrates and divided them into eight batches on average (six per batch). Then, with the MoS_2_ bulk sticking to the substrates with the tape, thermal treatment was carried out for 5 h at eight temperatures, from 35 to 140°C (at intervals of 15°C), corresponding to the eight batches of Si/SiO_2_ substrates with MoS_2_ bulk, as mentioned above. At the end of the thermal treatment, we obtained MoS_2_ nanoflakes in two ways, through direct or precooling exfoliation after heating. Each method was applied to three bulk-on-substrate samples per batch; see Figure 2A.

To clarify the effects of heating time on flake quality, we prepared 30 Si/SiO_2_ substrates and divided them into 10 batches (three per batch). Subsequently, the substrates were heated at 110°C for different periods of time, from 30 to 300 min, at intervals of 30 min, corresponding to the 10 batches, as shown in Figure 2B. After thermal treatment, we exfoliated the tapes from the wafer, followed by pre-cooling for 10 min.

The characterization of the samples above was conducted using various instruments, as described below. The spectra of the flakes were analyzed with Raman spectrometers (model: RENISHAW inVia) and photoluminescence spectrometers (model: Edinburgh FS5). The electrical performance of the MoS_2_-FET was tested using semiconductor parameter analyzers (model: Keithley 4200). The micro-images of the samples were taken with an optical microscope (model: LEIKA DM2700M) and an atomic force microscope (model: HITACHI-AFM5000II), and the contact angles of interfaces were measured using a contact angle meter (model: KINO SL200KS).

## Results and Discussion

### Statistical Analyses Based on Microimages

**Figure 3:**
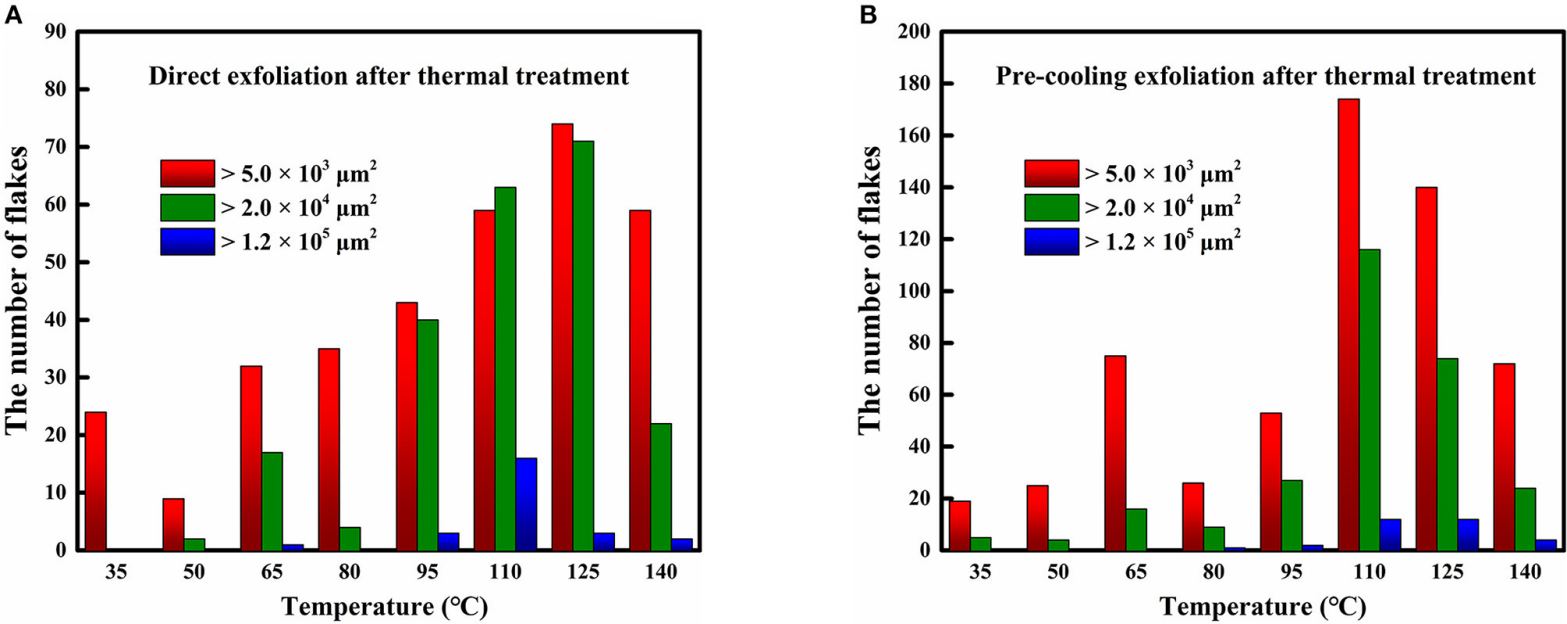
Quantity of certain MoS_2_ nanoflakes prepared by **(A)** direct and **(B)** pre-cooling exfoliation after thermal treatment for 5 h.

**Figure 4:**
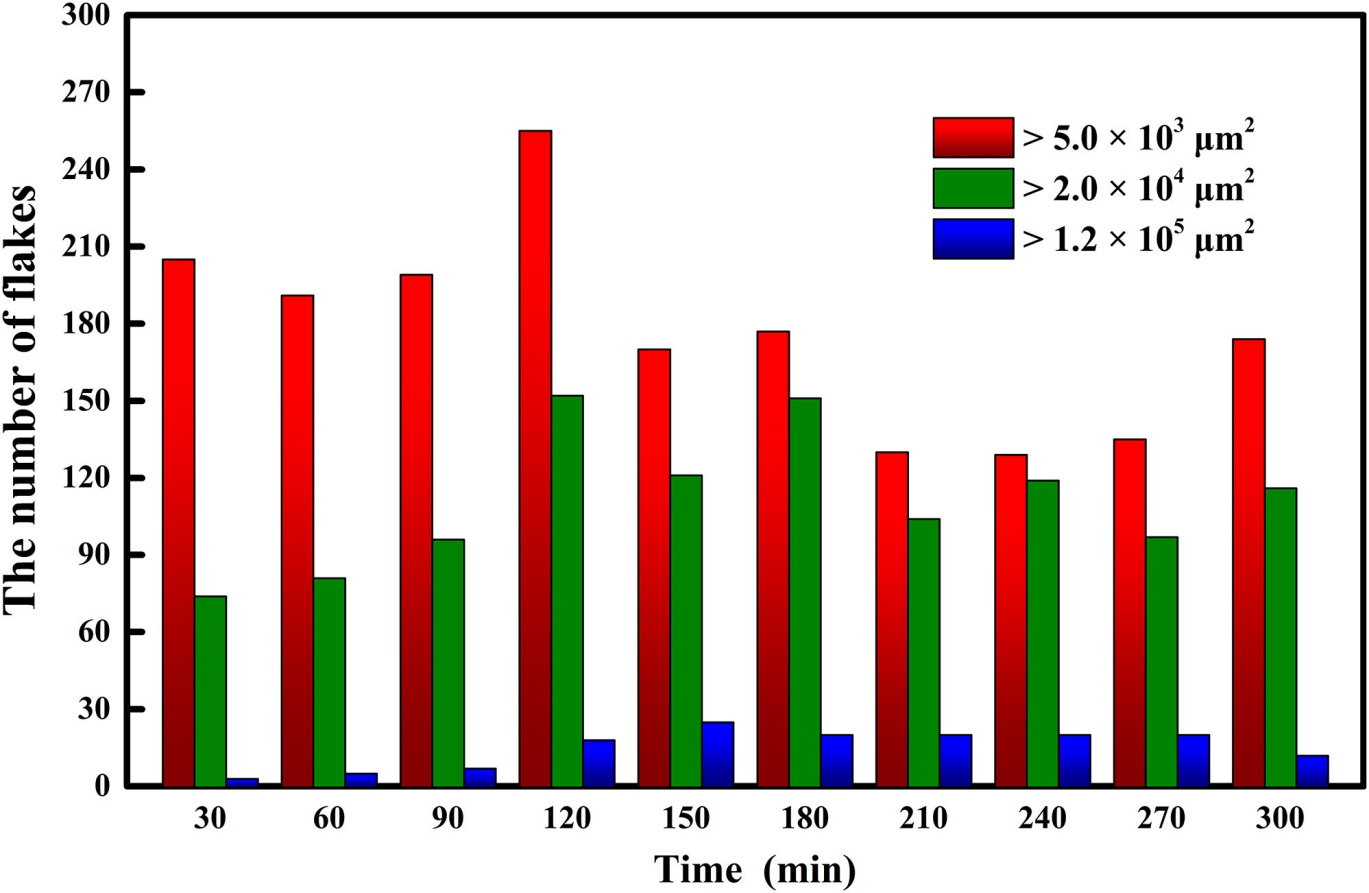
Statistics of MoS_2_ nanoflakes prepared with pre-cooling exfoliation after thermal treatment at different durations.

First, we observed the microimages of MoS_2_ nanoflakes with an optical microscope and counted the number of flakes with sizes of 1.2 × 10^5^, 2.0 × 10^4^, and 5.0 × 10^3^ μm^2^ on each substrate, corresponding to the temperature-dependent experiment (Figure 2A). As mentioned above, a batch of bulk-on-substrate samples were heated at temperatures from 35 to 140°C (increasing at intervals of 15°C) for 5 h. We then separated the samples in each batch into two halves. Three were exfoliated immediately, and the other three were separated after precooling for 10 min to obtain MoS_2_ flakes. The statistics for the average amount of flakes on the substrates for different heating temperatures are shown in Figures 3A,B. The values shown in the panels are relative to the results of direct or precooling exfoliation after heating, respectively. From these two figures, we found that the quantity of flakes that reaches a certain size was higher at 110°C in both direct and precooling exfoliation. The precooling method presented a greater number of MoS_2_ flakes than direct exfoliation. Thus, we concluded that exfoliation with precooling after heating at 110°C for 5 h is a suitable means of improving the yield of MoS_2_ flakes. Furthermore, according to the description given in section two, in the time-dependent experiment, 30 bulk-on-substrate samples were divided into 10 batches, with heating times from 30 to 300 min (increased by 30 min). The average amount for each batch of MoS_2_ nanoflakes reaching the sizes of 1.2 × 10^5^, 2.0 × 10^4^, and 5.0 × 10^3^ μm^2^ after precooling exfoliation at 110°C is presented in Figure 4. This shows that the size and yield of the flakes after heating for 120 min is generally high.

**Figure 5:**
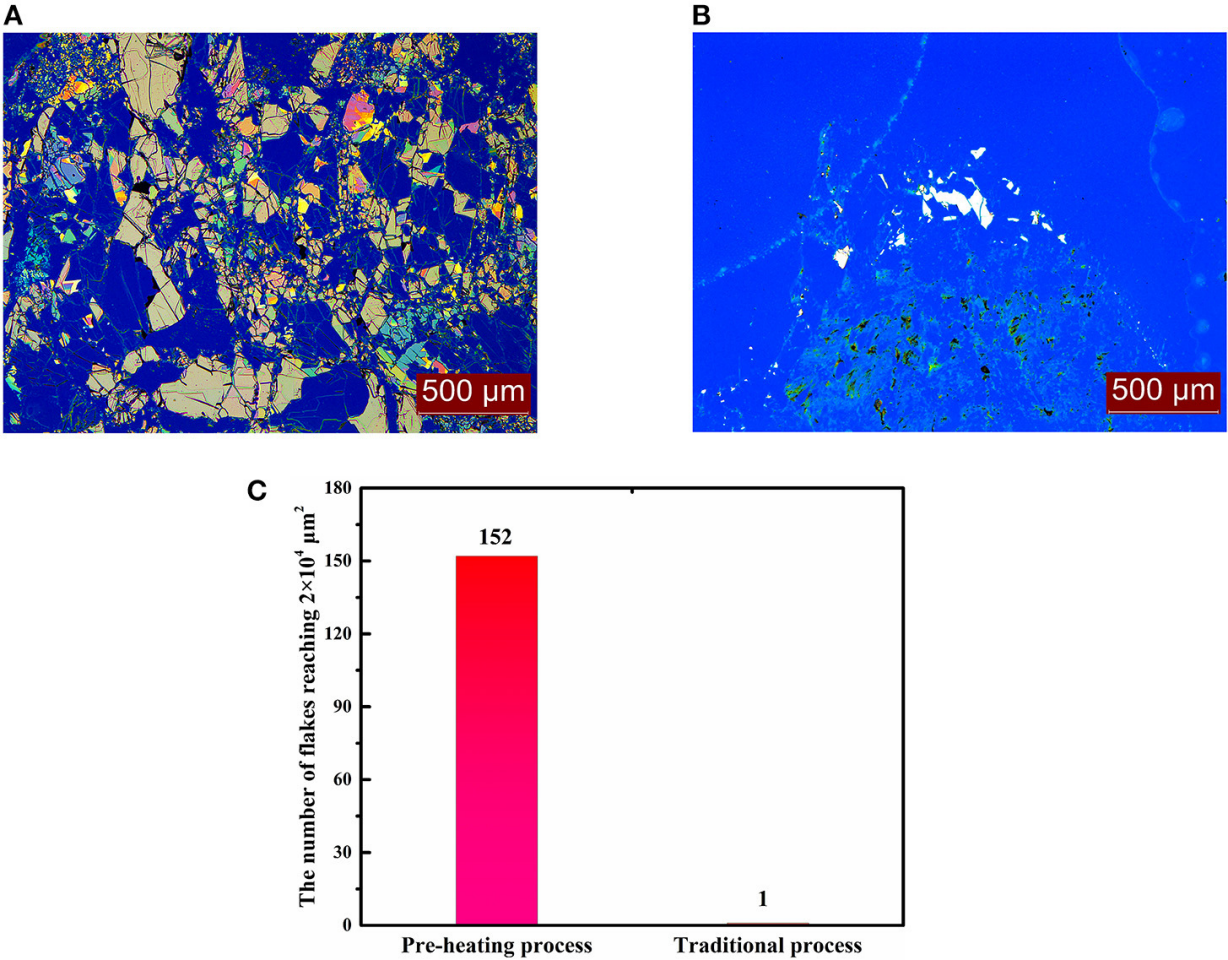
Microimages of MoS_2_ nanoflakes exfoliated by **(A)** pre-heating and **(B)** traditional processes and **(C)** their statistics.

**Figure 6:**
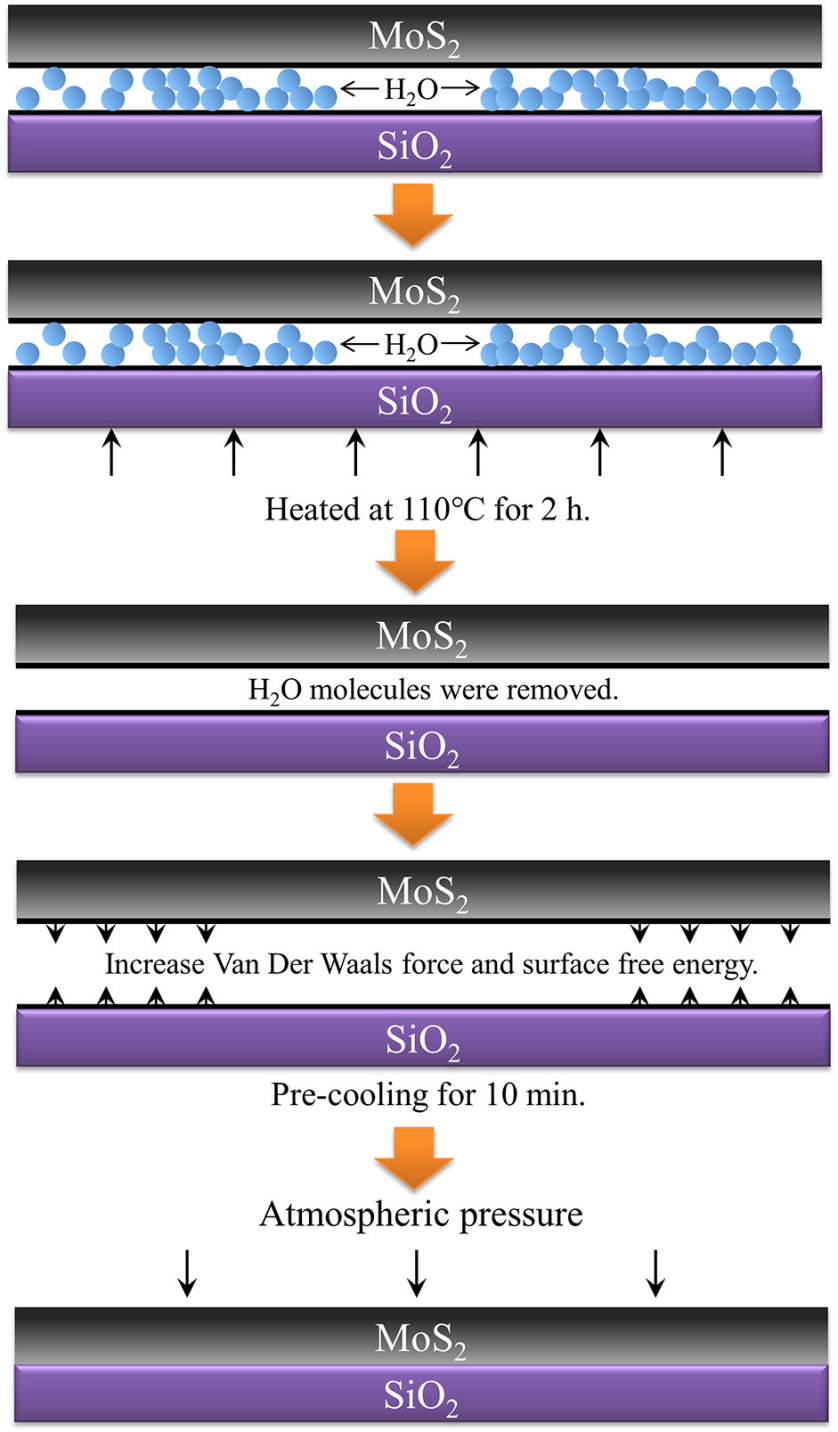
Mechanisms of exfoliation based on thermal treatment.

From the above phenomena, we can confirm that the most suitable way to prepare MoS_2_ nanoflakes in our experimental range is to treat the wafer with MoS_2_ bulk for 2 h at 110°C and then exfoliate the bulk after precooling for 10 min. A comparison with traditional exfoliation without any aid indicated that the size and yield of the flakes showed obvious improvement, as seen in Figures 5A,B. Statistical evaluation showed that through the heating process at 110°C for 2 h, 152 flakes were obtained that were larger than 2 × 10^4^ μm^2^, while only one was obtained through original mechanical exfoliation, as shown in Figure 5C. This characterization indicates that the quantity of flakes significantly improved in the thermal treatment process, compared with traditional mechanical exfoliation, which can be attributed to two factors, as shown in Figure 6.

First, heating at 110°C for 120 min leads to complete evaporation of the water between the MoS_2_ and the wafer. This mechanism plays a critical role in the adequate adhesion of the interface, with the help of atmospheric pressure. The van der Waals force of the interface may then be greater than the interlamination of the MoS_2_ bulk. In this way, the bulk can easily be dissociated into nanoflakes on the SiO_2_ substrate. It must be mentioned that the boiling point of water is generally considered to be 100°C, but considering the heat lost in thermal radiation and conduction, the actual heating temperature must be higher than 100°C and remain there for some time to ensure the complete evaporation of water at the interface. It can be shown by statistics that the quantity of MoS_2_ nanoflakes obtained at the heating temperature of 110°C is larger than that at 100°C.

Next, precooling before exfoliation may result in a difference in temperature between the surface and interior of the bulk. Higher temperatures cause a violent movement of molecules that may weaken the van der Waals force of the interlayer in the bulk. Furthermore, the van der Waals force between the MoS_2_ and the wafer is increased by the decreases in the temperature outside. Thus, a difference in van der Waals force appears, which is beneficial for the preparation of the MoS_2_ flakes through easier exfoliation from bulk.

The surface free energy (SFE) is a measure of the destruction of chemical bonds between molecules when creating a material surface. According to solid-state physical theory, surface atoms have more energy than internal atoms for the interruption of chemical bonds on the surface. According to the law of conservation of energy, a higher SFE of the substrate has the potential to be reduced through adsorption of other molecules or atoms around it. Here, the adsorption capacity of the solid surface to the materials in contact is improved (Dong et al., 2010). We tested the contact angle on the substrate treated at the temperatures 35, 50, 65, 80, 95, 110, 125, and 140°C. We then extracted the value of the SFE of substrates through a reformulation of the equation of state for the interfacial tensions proposed by Li and Neumann (1990) (see Equations 1 and 2), where γ_sl_, γ_sv_, and γ_lv_ represent the SFE of the solid and liquid interface, the solid SFE, and the liquid SFE, respectively; θ is the contact angle between the solid surface and liquid; and β is an empirical value, namely, 1.247 × 10^−4^. The calculated results are as shown in Table 1, and they demonstrate that the maximum SFE of the substrates occurs at the temperature of 110°C, which is consistent with our previous experiment. This indicates that thermal treatment at 110°C should also lead to an increase in SFE on the substrates, which further improves the quality of the exfoliated flakes.

(1) $$\begin{array}{l} \gamma_{sl}=\gamma_{lv}+\gamma_{sv}-2\sqrt{\left(\gamma_{lv}\gamma_{sv}\right)}e^{-\beta {\left(\gamma_{lv}-\gamma_{sv}\right)}^{2}} \end{array}$$

(2) $$\begin{array}{l} \cos\theta =-1+2\sqrt{\left(\frac{\gamma_{sv}}{\gamma_{lv}}\right)}e^{-\beta {\left(\gamma_{lv}-\gamma_{sv}\right)}^{2}} \end{array}$$

**Table 1:** Contact angle and corresponding SFEs of substrates heated to different temperatures.

**Temperature (^**°**^C)**	**35**	**50**	**65**	**80**	**95**	**110**	**125**	**140**
Contact angle (deg)	64.12	63.08	62.38	60.82	60.46	47.96	56.71	58.65
SFE of substrates (mJ/m^2^)	44.11	44.73	45.16	46.09	46.31	53.60	48.54	47.39

### Materials Characterization and Analyses

**Figure 7:**
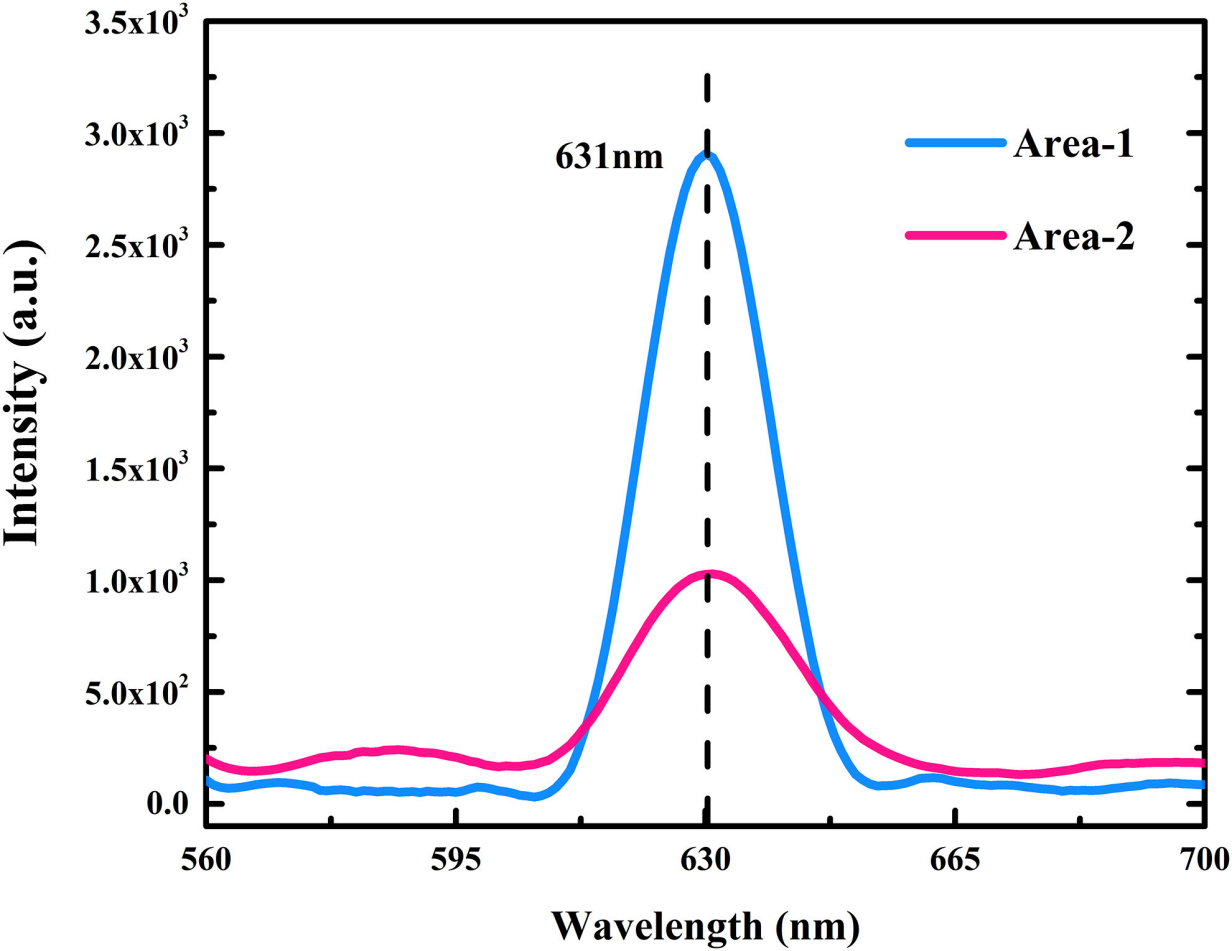
Photoluminescence spectrum of two test areas.

**Figure 8:**
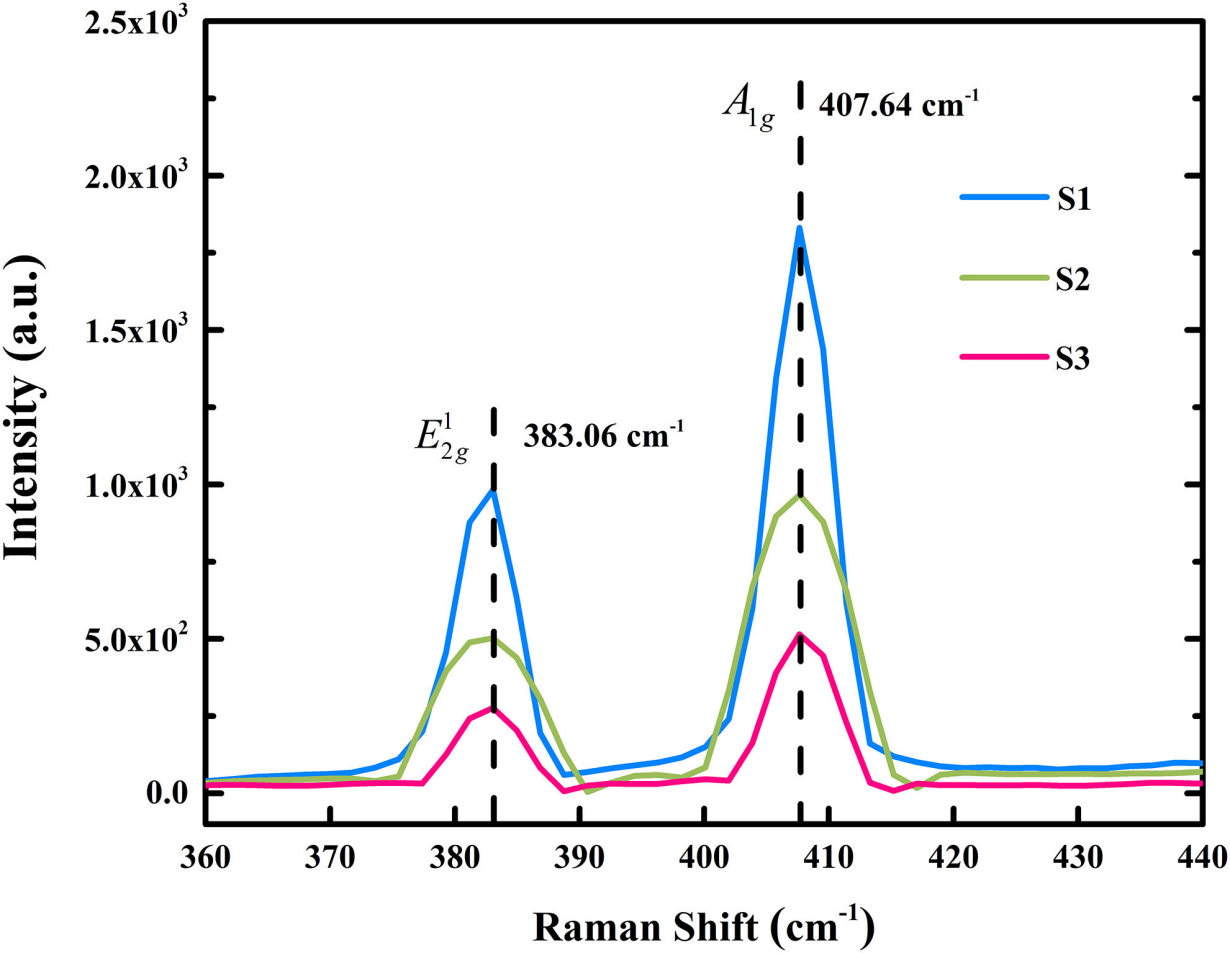
Raman spectrum of three MoS_2_ samples exfoliated using the new process.

**Figure 9:**
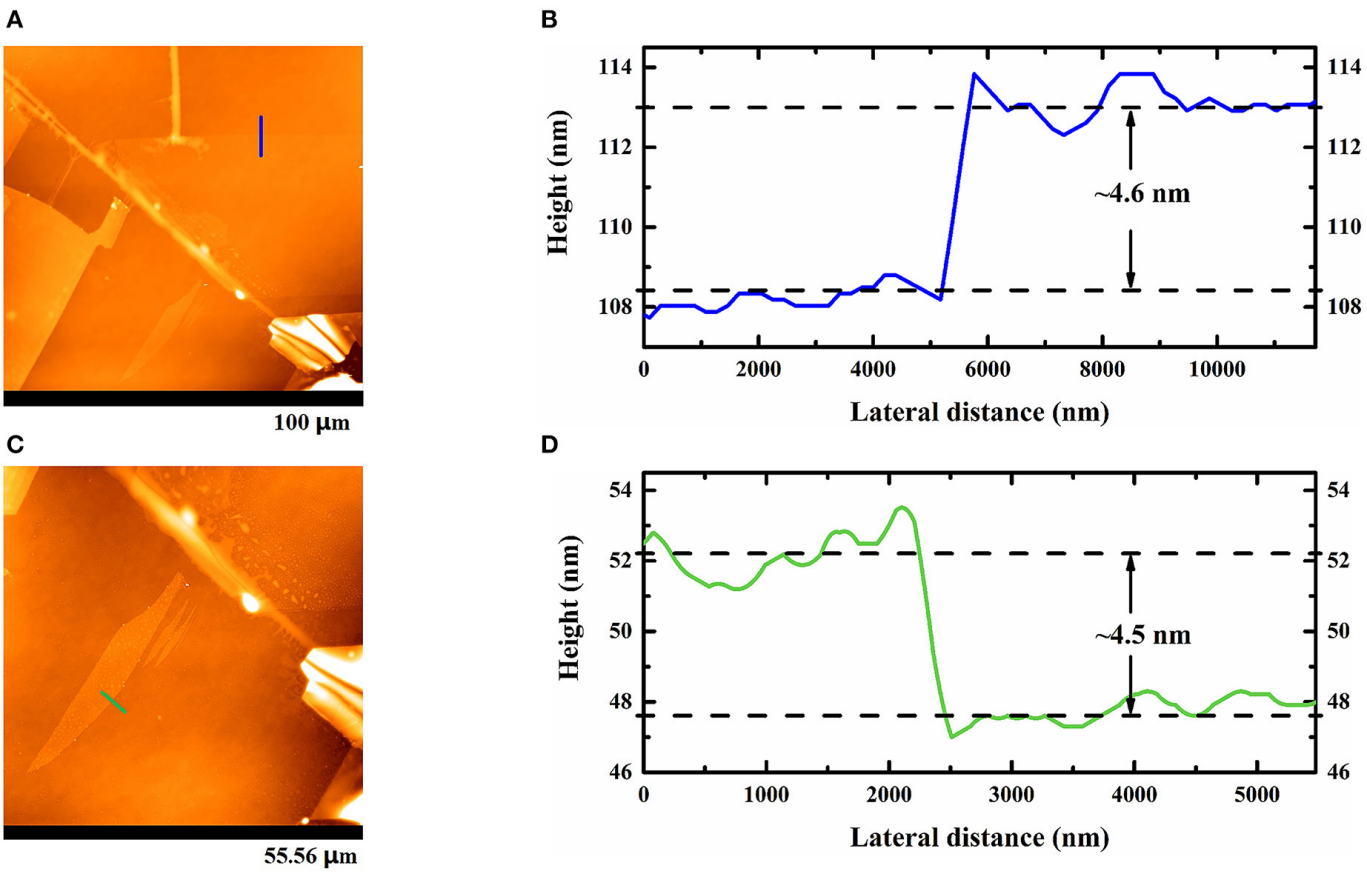
AFM images of three multilayer MoS_2_ nanoflakes **(A,C)** achieved with pre-heating and their corresponding height profiles, as shown in **(B,D)**.

To evaluate the results of the novel exfoliation process, material characterization was necessary. First, we observed the characteristic peaks of three MoS_2_ nanoflakes in a photoluminescence spectrum, as shown in Figure 7. We found that the peaks of two test areas were both 631 nm, which is close to the report of Splendiani et al. (2010). In addition, Raman spectra were used to confirm the thickness of the MoS_2_ flakes with respect to optical principle. Figure 8 shows the Raman spectrum of the sample excited by 532 nm laser line at room temperature. Both $E_{2g}^{1}$ and *A*_1*g*_ peaks in the spectrum were observed in three flakes. Overall, the differences in the two peaks were ~24.6 cm^−1^ at the test points. Thus, consulting Liang and Meunier (2014), we can confirm that all of the flakes were multilayered. Subsequently, the above analyses were further verified via AFM through a microimage of two MoS_2_ nanoflakes exfoliated after thermal treatment of the substrate, from which it was verified that the thicknesses of the flakes were 4.6 and 4.5 nm, respectively, as shown in Figure 9. Because the thickness of an MoS_2_ atomic layer is 0.65 nm, these flakes are about seven atomic layers thick. This is consistent with the features of multilayers, as confirmed by the Raman spectra (Liang and Meunier, 2014).

### Device Performance

**Figure 10:**
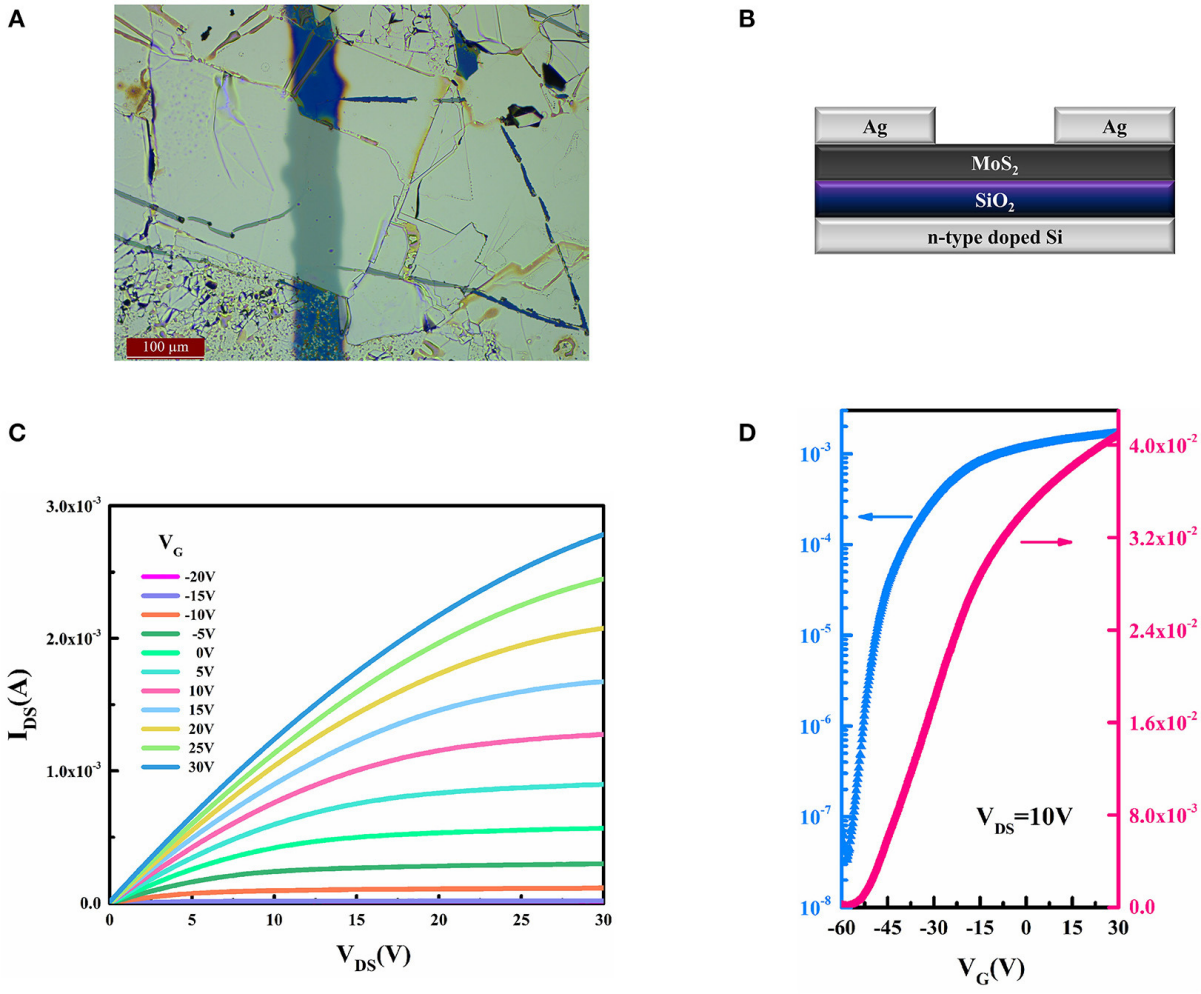
**(A)** Structure and **(B)** microimage of MoS_2_-FET and its **(C)** output curve and **(D)** transfer curve.

The characterization and discussion indicate that semiconducting devices can be produced more easily with the new process of thermal treatment outlined here, due to the high yield and big size of the MoS_2_ nanoflakes. Using an MoS_2_ nanoflake prepared by substrate heating, an FET was fabricated to assess the applicative effects of new processes. The device was built according to the so-called bottom-gate and top-contact structure, using Ag as a drain-source electrode and n-type-doped Si as a gate electrode. A structural sketch and a micro-image for FET are shown in Figures 10A,B, respectively. The output and transfer curves of the device are exhibited in Figures 10C,D, allowing us to ascertain that the saturated current in the channel is 1.28 × 10^3^ μA (*V*_G_ = 10 V). When *V*_DS_ = 10 V, the on/off ratio of *I*_DS_ in this device is ~10^5^, and the subthreshold swing is 317.46 mV/dec. Furthermore, due to the negative threshold voltage extracted from transfer curve, we can confirm that this device belongs to the depletion type. Additionally, the field-effect mobility of the device is 24.26 cm^2^/Vs, which is extracted with Equation 3, where μ_sat_ is the field-effect mobility in the saturated region of the device, *L* and *W* are the length and width of device channel, *I*_DS_ is the channel current of FET, *V*_G_ is the bias voltage between the gate and source electrode, and *C*_i_ is the typical capacitor value of SiO_2_ in the unit area as 11.5 nf/cm^2^. The electronic properties of the device were generally consistent with those found in an earlier work (Zhang et al., 2020), as shown in Table 2.

(3) $$\begin{array}{l} \mu_{sat}=\frac{2L}{WC_{i}}{\left[\frac{\partial \left(\sqrt{I_{DS}}\right)}{\partial V_{G}}\right]}^{2} \end{array}$$

**Table 2:** Performance of MoS_2_-FET using the new exfoliation process.

**Mobility (cm^**2**^/Vs)**	**Subthreshold swing (mV/dec)**	**Saturated I_**DS**_ (μA)**	**On/off ratio**
24.26	317.46	1.28 × 10^3^	5.0 × 10^4^

## Conclusion

We here report a simple way of improving the yield and size of MoS_2_ nanoflake preparation. Specifically, we exfoliate the samples with precooling after heating at 110°C for 5 h. The statistics on the microimages indicate that the quality of the flakes is obviously improved, which can be attributed to the differences in the van Der Waals force and the increase in SFE at the interface induced by thermal treatment. Using Raman and photoluminescence spectra, the multilayered structures of the MoS_2_ crystals are clarified. Following the new exfoliation process, an MoS_2_-FET was fabricated with an on/off current ratio of ~10^5^, a field-effect mobility of 24.26 cm^2^/Vs when *V*_DS_ = 10 V, and a saturated current of 1.28 × 10^3^ μA when *V*_G_ = 10 V, values that are generally consistent with those of devices reported previously. These results indicate that the method developed in this paper can efficiently prepare MoS_2_-FET and has a significant opportunity to become standard procedure for the preparation of 2D materials with an atomic layer structure.

## Data Availability Statement

The original contributions presented in the study are included in the article/supplementary material, further inquiries can be directed to the corresponding author/s.

## Author Contributions

YZ and XC: writing. SH, GZ, MZ, WQ, ZW, and XH: perform experiment. JW and HZ: supervision and review. All authors contributed to the article and approved the submitted version.

## Conflict of Interest

The authors declare that the research was conducted in the absence of any commercial or financial relationships that could be construed as a potential conflict of interest.
